# Interactions between latent variables in count regression models

**DOI:** 10.3758/s13428-024-02483-4

**Published:** 2024-08-26

**Authors:** Christoph Kiefer, Sarah Wilker, Axel Mayer

**Affiliations:** 1 Methods and Evaluation, Department of Psychology, Bielefeld University, Universitätsstraße 25, D-33501 Bielefeld, Germany; 2 Clinical Psychology and Psychotherapy, Department of Psychology, Bielefeld University, Universitätsstraße 25, D-33501 Bielefeld, Germany

**Keywords:** Latent interactions, Count outcomes, Poisson regression

## Abstract

In psychology and the social sciences, researchers often model count outcome variables accounting for latent predictors and their interactions. Even though neglecting measurement error in such count regression models (e.g., Poisson or negative binomial regression) can have unfavorable consequences like attenuation bias, such analyses are often carried out in the generalized linear model (GLM) framework using fallible covariates such as sum scores. An alternative is count regression models based on structural equation modeling, which allow to specify latent covariates and thereby account for measurement error. However, the issue of how and when to include interactions between latent covariates or between latent and manifest covariates is rarely discussed for count regression models. In this paper, we present a latent variable count regression model (LV-CRM) allowing for latent covariates as well as interactions among both latent and manifest covariates. We conducted three simulation studies, investigating the estimation accuracy of the LV-CRM and comparing it to GLM-based count regression models. Interestingly, we found that even in scenarios with high reliabilities, the regression coefficients from a GLM-based model can be severely biased. In contrast, even for moderate sample sizes, the LV-CRM provided virtually unbiased regression coefficients. Additionally, statistical inferences yielded mixed results for the GLM-based models (i.e., low coverage rates, but acceptable empirical detection rates), but were generally acceptable using the LV-CRM. We provide an applied example from clinical psychology illustrating how the LV-CRM framework can be used to model count regressions with latent interactions.

## Introduction

In psychology and the social sciences, researchers often model count outcomes accounting for latent predictors and their possible interactions. For example, Wilker et al. (2017) regressed symptom severity (i.e., how often did symptoms occur) of posttraumatic stress disorder on traumatic load, mental defeat, and their interaction. Both predictors were assessed using multiple items from psychometric questionnaires. Others studied the interactive effect of psychological distress and gender on problematic drinking behavior (i.e., number of alcoholic drinks; Rodriguez, Litt, & Stewart, 2020) or of callous traits and gender on antisocial outcomes (e.g., number of arrests; McMahon, Witkiewitz, Kotler, & The Conduct Problems Prevention Research Group, 2010.

Such analyses often apply a generalized linear model (GLM; McCullagh & Nelder, 1998) using fallible predictors such as a sum score. Prominent options for count outcomes are Poisson or negative binomial regression (Hilbe, 2011). These are GLMs with a logarithmic link function and a Poisson or negative binomial distributed random component, which take the discrete and non-negative nature of count outcomes into account. The predictors are assumed to be observed without error and fixed by design, which is often not plausible for psychological measurements such as test scores.

While it seems to be a wide-spread approach to neglect measurement error in such analyses (Cheung, Cooper-Thomas, Lau, & Wang, 2021; Cortina, Markell-Goldstein, Green, & Chang, 2021), it is well known to have unfavorable consequences in GLMs: First, measurement error typically attenuates the regression coefficients towards zero, but in settings with multiple error-prone predictors both over- and underestimation can occur (Carroll, Ruppert, Stefanski, & Crainiceanu, 2006; Kiefer, & Mayer, 2021a). Thus, attenuation bias complicates the identification of relevant product terms. Second, the reliability of the product term of two variables depends on their respective reliabilities and is typically lower than either of these (Bohrnstedt & Marwell, 1978; Busemeyer & Jones, 1983). Thus, products of fallible predictors strongly contribute to attenuation bias in parameter estimation. Consequently, there is a need for count regression models accounting for latent predictors and their (latent) interactions.

While latent interaction models with continuous outcomes (e.g., Kelava et al., 2011; Klein & Moosbrugger, 2000) with possible extensions for non-normally distributed latent variable indicators (e.g., Jin, Vegelius, & Yang-Wallentin, 2020) are well understood, latent interactions in count regression models are rarely discussed. A notable exception is the negative binomial multigroup structural equation model (NB-MG-SEM) by Kiefer and Mayer (2021a, 2021b). The NB-MG-SEM allows interactions between latent continuous predictors and manifest categorical predictors by using a multigroup SEM approach. Recently, Rockwood (2021) proposed a generalized structural equation model (G-SEM) which can be used for the estimation of count regression models with latent predictors. While the G-SEM framework is very versatile, the formulation and implementation of Rockwood (2021) does not include product terms of the latent predictors.

In this paper, we contribute to the literature on latent interaction models for count outcomes in three ways: First, we present a general framework for a latent variable count regression model (LV-CRM) allowing for latent interactions. This framework is derived as extension of a GLM and also builds on the G-SEM framework by Rockwood (2021). Second, in three Monte Carlo simulation studies we compare the estimation accuracy of the proposed approach to GLM-based count regression models. Third, we provide an empirical example from clinical psychology to illustrate how the LV-CRM can be used to model count regressions with latent interactions in applied research.

## Generalized linear models for count outcomes

In the following, we derive the LV-CRM as an extension of the GLM because many applied researchers are familiar with the GLM notation as well as GLM-based count regression models like Poisson or negative binomial regression. We start with describing the core elements of a GLM-based Poisson regression model, explain how interactions can be included within a GLM, and discuss the impact of measurement error in the predictors on the parameter estimation. In the next section, we introduce the LV-CRM as extension of the GLM allowing for latent covariates and latent interactions.

GLMs have been proposed by Nelder and Wedderburn (1972) and it can be shown that several well-known regression models, as for instance, the logistic regression model or the general linear model, are special cases of the GLM (McCullagh & Nelder, 1998). The key idea is that all these regression models can be decomposed into three main components: (a) a *random component* describing the conditional distribution of the outcome variable; (b) a weighted linear combination of the predictor variables (i.e., the *linear predictor*), and; (c) a functional connection between the two, called the *link function*.

In principle, each component can be modified independently from the other two, which results in a very flexible way to model regressive dependencies among manifest variables.

**Fig. 1:**
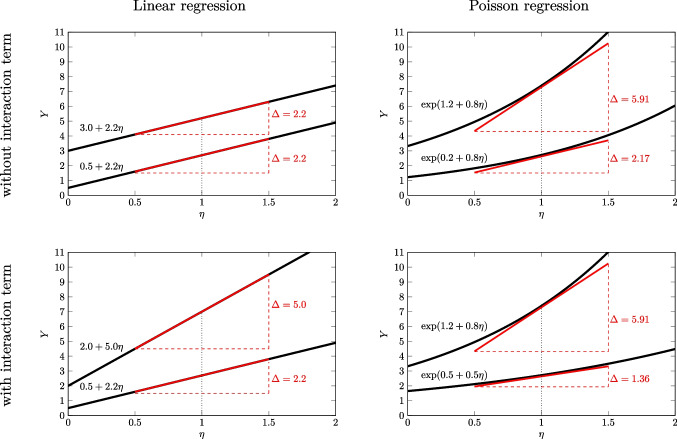
Illustration of the interaction effects in linear and Poisson regression models without and with a dedicated interaction term. *Red lines* reflect linear approximations of the regression lines at $\eta =1$ and $\Delta$ indicates the slope of these approximations

### Poisson regression model

GLMs for count outcomes are referred to as the family of Poisson regression models (e.g., Coxe, West, & Aiken, 2009). The standard Poisson regression model, while being parsimonious and comprehensible, is usually not suitable in applied scenarios. Thus, alternatives as the negative binomial regression are also part of the family of Poisson regressions. For a gentle introduction to Poisson regression models, see Coxe et al. (2009).

Consider a vector of *N* i.i.d. sampled outcome variables $\varvec{y}= (Y_1,\dots ,Y_N)'$, where the index $i=1,\dots ,N$ indicates the individual observations, and for each individual the observation of *m* fixed predictor values $\varvec{z}_i = (1, z_{1i}, \dots , z_{mi})$ with index $j=1,\dots ,m$. Then, according to McCullagh and Nelder (1998), the GLM-formulation of a standard Poisson reg-ression model involves the following three main components:

(a) the random component is Poisson distributed, $Y_i \sim \mathcal {P}(\mu _{Y_i})$, that is, we consider each observation $Y_i$ of our count outcome to follow a Poisson distribution with expectation $\text {E}(Y_i) = \mu _{Y_i}$. The Poisson distribution comes with the property of equidispersion, meaning that the variance and the expectation of $Y_i$ are identical. However, researchers often encounter overdispersed count outcomes, that is, the variance of $Y_i$ exceeds its mean. In this case, an additional variance component can be introduced to the model, leading to Poisson-mixture distributions such as the negative binomial distribution (i.e., a Poisson-gamma mixture; Hilbe, 2011) or the Poisson-lognormal (PLN) distribution (Bulmer, 1974).

(b) The linear predictor $\pi _i$ is defined as a weighted linear combination of the predictors $\varvec{z}_i$, where the weights $\varvec{\beta } = (\beta _0,\beta _1,\dots ,\beta _m)'$ are called regression coefficients:

$$\begin{aligned} \pi _{i} = \beta _{0} + \sum _{j=1}^{m} \beta _{j} \cdot z_{ji} = \varvec{z}_{i} \varvec{\beta } \end{aligned}$$

Below, we show how interactions between two or more predictors can be included in a GLM. It is important to note that the $z_{ji}$ are treated as fixed constants. As a consequence, they are treated as perfectly reliable measures. However, this is not plausible if fallible scores of latent constructs (e.g., test scores from an intelligence test) are used as predictors, which can lead to attenuation bias in the estimated regression coefficients.

(c) For count outcomes, the expectation of the outcome variable $\mu _{Y_i}$ and the linear predictor $\pi _i$ are commonly connected via a logarithmic link function (short: log link), that is,

$$\begin{aligned} \log (\mu _{Y_i}) = \pi _i \, \Leftrightarrow \, \mu _{Y_i} = \exp (\pi _i) \end{aligned}$$

which naturally accounts for the lower bound of count outcomes at zero.

**Fig. 2:**
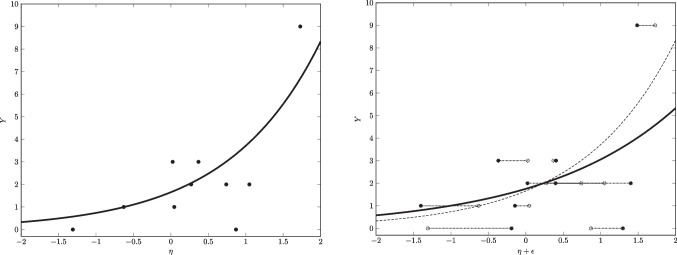
*Left panel:* Poisson regression (*black line*) of *Y* on the true scores of a predictor variable $\eta$ (*black dots*). *Right panel:* Poisson regression (*black line*) of *Y* on fallible scores of the predictor variable (i.e., $\eta$ plus a measurement error $\epsilon$; *black dots*). *Dashed regression lines* reflect deviations from the Poisson regression with true scores

Estimation of a Poisson regression model can be done via iteratively weighted least squares to find the maximum likelihood estimates and the corresponding standard errors. For more details, see Hilbe (2011, Ch. 4)

### Interactions in Poisson regression models

Whenever the effect of one predictor depends on the values of another, we can model this using product terms. In the GLM framework, product terms of the observed variables can be added as a new variable to the linear predictor, e.g., $z_{3i} := z_{1i} \cdot z_{2i}$. In a simple example with only two covariates and their interaction, the equation for the linear predictor is:

$$\begin{aligned} \pi _i = \beta _0 + \beta _1 z_{1i} + \beta _2 z_{2i} + \beta _3 \cdot \underbrace{z_{3i}}_{= z_{1i} \cdot z_{2i}} \end{aligned}$$

If, for instance, $z_2$ is a binary trauma variable (e.g., $z_2=1$: trauma experienced vs. $z_2=0$ not experienced) and $z_1$ is age, we can compute the conditional regression of the count outcome on age given values of the trauma variable:

$$\begin{aligned} \pi _i&= \beta _0 + \beta _1 z_{1i}&\qquad&\text {for } z_2=0 \\ \pi _i&= (\beta _0 + \beta _2) + (\beta _1 + \beta _3) \cdot z_{1i}&\qquad&\text {for } z_2=1 \end{aligned}$$

The first equation represents the relationship between the outcome *Y* and the predictor $z_1$ (i.e., age) if a trauma was not experienced ($z_2=0$), and the second equation if a trauma was experienced, respectively.

It is important to note that Poisson regression models – just like other non-linear models – can contain interaction effects even if the coefficient of the product term is zero, that is, $\beta _3=0$. This phenomenon is called *natural* (or sometimes: model-inherent) interaction (Karaca-Mandic, Norton, & Dowd, 2012; McCabe, Halvorson, King, Cao, & Kim, 2022) and it is illustrated in Fig. 1. In the upper right panel, the coefficient of $z_1$ is identical for both $z_2=0$ and $z_2=1$. However, for someone with a value of $z_1=1$ the slope (as indicated by the red lines) varies considerably depending on the moderator. This is in contrast to the linear model (upper left panel) where the absence of a product term implies parallel lines. Thus, a product term can add to the complexity of the interaction pattern in a Poisson regression (lower right panel), but its absence is not equivalent to the absence of interaction.

The (linear) slopes of $z_1$, which are illustrated with the red lines in Fig. 1, are called *marginal effects* and they are defined as the first derivative of the regression function. The effect of a third variable (i.e., $z_2$) on the marginal effects of $z_1$, can then be defined as second-order mixed partial derivative (Kim & McCabe, 2022):

1 $$\begin{aligned} \zeta _{jk} := \frac{\partial ^2 \exp (\pi )}{\partial z_j \partial z_k} \end{aligned}$$

This definition of an interactive effect has two important properties: First, $\zeta _{ijk}$ can be non-zero even if product terms are excluded (or $\beta _3=0$). Second, $\zeta _{jk}$ varies among individuals and is not a constant. That is, interaction effects vary as function of the predictors both involved and not involved in a product term.

Kim and McCabe (2022) propose three approaches to summarize and report the interaction effects $\zeta _{jk}$: First, it is possible to plug-in the observed predictor values and then compute summary statistics of the individual interaction effects, for example, an average interaction effect. Second, $\zeta _{jk}$ is computed for representative points in the sample, for example, an “average” person (i.e., at the means of all covariates) and one standard deviation above and below this average. We will illustrate this approach in our empirical example below. Third, $\zeta _{jk}$ is computed at substantively relevant points, for example, at a specific cutoff. It is possible to obtain standard errors for the interaction effects $\zeta _{jk}$ by using the Delta method (Raykov & Marcoulides, 2004). Note, however, that if the interaction effects are computed at values estimated from the sample (e.g., sample mean), their sampling variance also has to be taken into account for reliable inferences (Liu, West, Levy, & Aiken, 2017).

**Table 1:** Overview and model comparison

	GLM	LV-CRM
Mean model:	$\text {E}(Y_i)$ $= \mu _{Y_i}$	$\text {E}(Y_i|\varvec{\eta }_i)$ $= \mu _{Y_i}$
Linear predictor:	$\pi _i$ $= \varvec{z}_{i} \varvec{\beta }$	$\pi _{i}$ $= \varvec{z}_{i} \varvec{\beta } + \varvec{\Gamma }_{1} \varvec{\eta }_{i} + \varvec{\eta }_i' \varvec{\Gamma }_{2} \varvec{\eta }_i + \varvec{\eta }_i' \varvec{\Omega }_2 \varvec{z}_i$
Link function:	$\mu _{Y_i}$ $= \exp (\pi _i)$	$\mu _{Y_i}$ $= \exp (\pi _i)$
Measurement model:		$\varvec{w}_i$ $= \varvec{\nu } + \varvec{\Lambda } \varvec{\eta }_i + \varvec{\epsilon }_i$

### Measurement error in the covariates

As stated before, a GLM assumes fixed predictors, which (a) are perfectly reliable and (b) do not vary from one sample to another. In psychological research, this is often not a realistic assumption, especially if predictors are randomly sampled, fallible measures of unobservable constructs (e.g., motivation, intelligence). If random measurement error in predictors is ignored, the regression coefficients get attenuated towards zero. This phenomenon known as attenuation bias (Carroll et al., 2006) is illustrated in Fig. 2. While the left panel shows a Poisson regression based on the true values of a covariate $\eta$, the right panel shows the attenuation of the regression line as an effect of measurement error $\epsilon$ added to the covariate. While attenuation bias is usually associated with attenuation towards zero, biases in all directions can be observed with multiple fallible covariates (Kiefer & Mayer, 2021a; Carroll et al., 2006).

In the literature, several approaches have been proposed dealing with measurement error in covariates. Some approaches assume that the distribution of the latent variables is known (e.g., normally distributed with known mean and variance) and use this information to adjust the estimates of the regression coefficient (Guo & Li, 2002; Kukush, Schneeweis, & Wolf, 2004). Other approaches include a measurement model to estimate the distribution of the latent variables (Carroll et al., 2006; Skrondal & Kuha, 2012). These later approaches are called *regression calibration*. The measurement model is either included directly into a joint estimation with the regression coefficients or estimated first in a two-step procedure, where then distributional parameters or factor scores are used in the regression estimation (for more information on the two-step procedure, see Rosseel & Loh, 2022). However, these approaches are rarely extended to scenarios with product terms in non-linear models, with some exceptions treating this issue for logistic regression models (e.g., Carroll et al., 2006, p.165). We are not aware of any contribution specifically addressing such adjustments focusing on both Poisson regressions and product terms.

Attenuation bias affects fallible predictors in general, but is likely to be exacerbated when product terms from fallible predictors are involved. This is because the reliability of the product term is usually lower than that of either of the interacting variables (Busemeyer & Jones, 1983). Bohrnstedt and Marwell (1978) show that for two predictors with reliability of .8, the reliability of the product term can drop below .6 (depending on their scaling and correlation). Nevertheless, it still seems to be a widespread approach to neglect measurement error in regression analyses containing interactions (Cheung et al., 2021; Cortina et al., 2021).

## Latent variable count regression model

In this section, we introduce a latent variable count regression model (LV-CRM) framework for count regression models involving latent predictors, their interactions, manifest predictors, and possible latent-manifest interactions. For didactic reasons, we show how the LV-CRM can be derived as an extension of the GLM and therefore also stick to the common notation of the three main parts of the GLM.

We consider two steps to extend a GLM to a LV-CRM: (a) adding a measurement model as fourth model component and second, allowing latent variables, their interactions, and interactions between latent and manifest predictors as part of the linear predictor. Table 1 provides an overview and comparison of the GLM and the LV-CRM.

Note that the LV-CRM overlaps with the G-SEM framework (Rockwood, 2021), which, however, does not allow for interaction terms involving latent variables. The LV-CRM can be estimated with general purpose statistical software, for example, Mplus (Muthén & Muthén, 1998-2017), the GLLAMM approach in Stata (Skrondal & Rabe-Hesketh, 2004), or Stan (Stan Development Team, 2024). However, to our knowledge, the LV-CRM has not been previously described in the literature nor is there a technical documentation of how the LV-CRM is implemented, for example, in Mplus. Below, we will provide Mplus syntax for the empirical example as well as an open-source implementation in R.

### Measurement model

In psychology and the social sciences, explicitly modeling measurement error and latent variables using a common factor technique (Bollen, 1989) is a popular approach. The key idea is that we have *q* measurements $\varvec{w}_i=(W_{1i},\dots ,W_{q})'$ (e.g., items) intended to measure (multiple) latent variables (e.g., intelligence) and common factors $\varvec{\eta }_i=(\eta _{1i},\ldots ,\eta _{pi})'$ with $p \le q$ are introduced to model the correlations among the measurements:

$$\begin{aligned} \varvec{w}_i = \varvec{\nu } + \varvec{\Lambda } \varvec{\eta }_i + \varvec{\epsilon }_i \end{aligned}$$

where $\varvec{\nu }$ is a $q \times 1$ vector of intercepts; $\varvec{\Lambda }$ is a $q \times p$ matrix of factor loadings; and $\varvec{\epsilon }_i$ is a $q \times 1$ vector of measurement error variables. The observed indicators $\varvec{w}_i$ are represented by a linear function of the latent variable plus measurement error. We assume that the measurement error variables $\varvec{\epsilon }_i=(\epsilon _{1i},\ldots ,\epsilon _{qi})$ as well as the latent variables $\varvec{\eta }_i$ are multivariate normally distributed with $\varvec{\epsilon }_i \sim \mathcal {N}(\textbf{0}, \varvec{\Theta })$ and $\varvec{\eta }_i \sim \mathcal {N}(\varvec{\mu }_{\eta }, \varvec{\Sigma }_{\eta })$. Latent variables and measurement errors are independent from each other.

Identification of the model can be achieved through standard identification rules for structural equation models. That is, the scale of the latent variables has to be specified. Two popular methods for scaling the latent variables are (a) fixing one loading to one and one intercept to zero (typically for the first indicator) or (b) fixing the mean and variance of the latent variable to 0 and 1, respectively (cf. Kline & Little, 2023). Different scaling methods lead to equivalent models (i.e., point estimates are algebraic transformations of each other), but statistical inferences based on the Wald test can vary among scaling methods (Klopp, & Klößner, 2021).

### Latent predictors and interactions

Now, we add the latent variables defined in the measurement model, and their possible interactions to the linear predictor component of the LV-CRM:

$$\begin{aligned} \pi _{i}&= \underbrace{\beta _{0} + \sum _{j=1}^{m} \beta _{j} \cdot z_{ji}}_{\text {GLM}} + \underbrace{\sum _{k=1}^{p} \gamma _{k} \cdot \eta _{ki}}_{\text {latent predictors}} + \underbrace{\sum _{k=1}^p \sum _{l=k+1}^{p} \gamma _{kl} \cdot \eta _{ki} \cdot \eta _{li}}_{\text {latent interactions}}\\&\quad + \underbrace{\sum _{j=1}^m \sum _{k=1}^{p} \omega _{jk} \cdot z_{ji} \cdot \eta _{ki}}_{\text {latent-manifest interactions}} \end{aligned}$$

As denoted in the braces, the first part is equivalent to the linear predictor of the GLM. By adding the latent variables with regression coefficients $\gamma _k$ to the predictor (i.e., the second part), we obtain a special case of the G-SEM (Rockwood, 2021). The third part adds latent interactions with regression coefficients $\gamma _{kl}$ to the linear predictor^1^. The fourth part allows for interactions between latent and observed predictors with regression coefficients $\omega _{jk}$. Of course, some of the regression coefficients can be zero leading to more parsimonious models. The linear predictor in matrix notation is:

$$\begin{aligned} \pi _{i} = \varvec{z}_{i} \varvec{\beta } + \varvec{\gamma }' \varvec{\eta }_{i} + \varvec{\eta }_i' \varvec{\Gamma } \varvec{\eta }_i + \varvec{\eta }_i' \varvec{\Omega } \varvec{z}_i \end{aligned}$$

where $\varvec{\gamma }$ is a $p \times 1$ vector of regression coefficients; $\varvec{\Gamma }$ is a $p \times p$ upper triagonal matrix of regression coefficients; $\varvec{\Omega }$ is a $p \times m$ matrix of regression coefficients. Conceptually, the LV-CRM belongs to the regression calibration approaches mentioned earlier (Carroll et al., 2006; Skrondal & Kuha, 2012).

In the LV-CRM, the product terms are identified if the latent variables are identified. There are no additional assumptions or specifications necessary. This is achieved through the specification on an individual level: If the individual values of the latent variables are identified, then the individual product terms are also identified. Note that different estimation methods will approach this specification differently. For example, in a Bayesian framework, individual values of the latent variables will be sampled and directly plugged in the formulas. In contrast, the marginal maximum likelihood approach will integrate the latent variables out of the individual likelihood function. The next section provides an overview of the marginal maximum likelihood estimation.

### Parameter estimation and standard errors

In this section, we provide a brief overview of maximum likelihood estimation of the proposed model. The overview is meant to give some intuition on the estimation technique and why no additional measurement models (e.g., as in a product-indicator approach; Kenny & Judd, 1984) or distributional assumptions (e.g., as in the LMS approach; Klein & Moosbrugger, 2000) are required for identification of the product terms. For a comprehensive illustration of the marginal likelihood technique and possible implementations, see Rockwood (2021) or Skrondal and Rabe-Hesketh (2004, Ch. 6). Alternative estimation methods for the LV-CRM exist, for instance, using an EM algorithm as in Mplus (Muthén & Muthén, 1998-2017) or Bayesian methods (Asparouhov & Muthén, 2021; Stan Development Team, 2024).

**Fig. 3:**
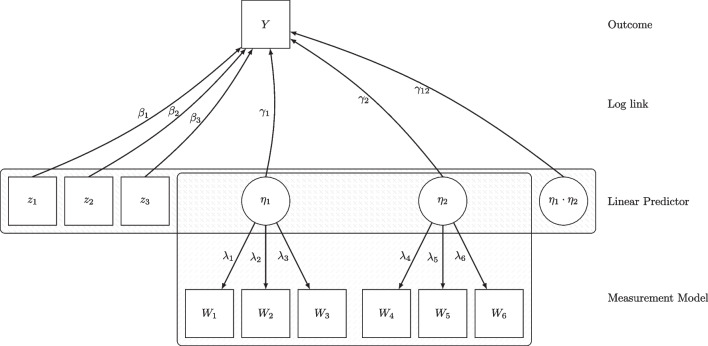
Path model depicting the four components of a LV-CRM. Example shows the Poisson regression of outcome variable *Y* on the manifest predictors $z_1$ to $z_3$, the latent predictors $\eta _1$ and $\eta _2$, and their interaction term $\eta _1 \cdot \eta _2$

For didactic reasons, we will restrict ourselves to describing the maximum likelihood estimation for the model depicted in Fig. 3. This is actually the model used for simulation study 2 and very similar to the empirical example. The case-wise log-likelihood function for this model can be written as:

$$\begin{aligned} \mathcal {L}_i(\varvec{\theta }|y_i, \varvec{z}_i, \varvec{w}_i)= &  \int _{\eta _1} \int _{\eta _2} f(y_i|\varvec{z}_i, \eta _1, \eta _2) \cdot f(\varvec{w}_i | \eta _1, \eta _2)\\ &  \cdot f(\eta _1, \eta _2) \, d(\eta _1, \eta _2) \end{aligned}$$

where $$y_i$$, $$\varvec{z}_i = (z_{1i}, z_{2i}, z_{3i})$$, and $$\varvec{w}_i = (W_{1i}, W_{2i}, W_{3i},$$$$ W_{4i}, W_{5i}, W_{6i},)$$ are the individual values of the observed variables. Since the values of the latent variables $$\eta _1$$ and $$\eta _2$$ are not observed, they are integrated out.

There is no closed-form solution for the log-likelihood function and hence it has to be approximated through numerical techniques:

$$\begin{aligned} \mathcal {L}_i(\varvec{\theta }|y_i, \varvec{z}_i, \varvec{w}_i) \approx \sum _{j=1}^M \omega _{j} \cdot f(y_i|\varvec{z}_i, \eta _{1j}^*, \eta _{2j}^*) \cdot f(\varvec{w}_i | \eta _{1j}^*, \eta _{2j}^*) \end{aligned}$$

where *M* is the number of integration points, $\omega _j$ is an integ-ration weight, and $\eta _{1j}^*$ and $\eta _{2j}^*$ are the integration points, respectively. An advantage of this procedure is that it provides a fixed set of values for the latent variables for each person in each iteration. Similar to the procedure in a GLM, we can use these latent variable values to compute product terms within the linear predictor. This is why the latent interaction term is presented as part of the linear predictor in Fig. 3, but not as part of the measurement model. The product term is only part of the linear predictor and the linear predictor is only part of the density function of the outcome variable $f(y_i|\varvec{z}_i, \eta _{1j}, \eta _{2j})$. Now, for each part sum of the approximated case-wise likelihood, we can simply compute the linear predictor $\pi _i(j)$ as a function of the integration points:

$$\begin{aligned} \pi _i(j) =&\beta _0 + \beta _1 \cdot z_{1i} + \beta _2 \cdot z_{2i} + \beta _3 \cdot z_{3i} + \gamma _1 \cdot \eta _{1j}^*\\&+ \gamma _2 \cdot \eta _{2j}^* + \gamma _{12} \cdot \eta _{1j}^* \cdot \eta _{2j}^* \end{aligned}$$

These models can become computationally demanding if the number of latent variables and thus the integration points rises, and we will discuss some approaches to reduce the computational burden. Standard errors can be derived using standard maximum likelihood theory, but this step is also computationally demanding as the second order derivatives of the log-likelihood have to be numerically approximated, too.

## Simulation studies

We conducted three Monte Carlo simulation studies to examine the performance of the LV-CRM framework under various empirical conditions and compared it to GLM-based Poisson or negative binomial regressions. From a substantive point of view, it is most interesting under which conditions the potential gains from the LV-CRM (e.g., reducing attenuation bias, increasing power) outweigh the costs (e.g., additional distributional assumptions, potential bias and numerical instability for insufficient sample sizes). Thus, we aligned our simulation studies with the aim to provide guidelines for substantive researchers which model to prefer under which conditions. The first simulation study focused on the extent of attenuation bias in a Poisson regression model with two latent variables and their interaction. It examines the question of how much bias one can expect given certain reliabilities of the sum scores, while still being computationally feasible to replicate by the interested reader. The second simulation study focused on two questions, namely, (a) if and how much attenuation bias can spill over to regression coefficients of perfectly reliable measures, and (b) how the statistical inferences from the LV-CRM perform and how they compare to GLM-based inferences. As this simulation study includes standard error estimation for the LV-CRM, it is computationally more burdensome. The third simulation study focused on attenuation bias in more complex scenarios, where we considered three latent variables and their two-fold interactions. We examined attenuation bias for different combinations of product term coefficients as well as correlational patterns among the latent variables. Due to the required three-dimensional numerical integration, this simulation was computationally demanding, too. The corresponding R code as well as the final results for all three simulation studies are available from OSF via https://osf.io/q7knc.

### Simulation study 1

The main focus of our first simulation study is to investigate the effect of different magnitudes of reliability of the fallible predictors on attenuation bias and how well the LV-CRM can de-attenuate the estimated regression coefficients. We pursue this focus with a small-scale simulation that can be reproduced by the interested reader within reasonable time. In the simulation studies 2 and 3, we will investigate additional aspects of statistical inferences and higher-dimensional numerical integration which are computationally more demanding. Final results for all three simulation studies are included in the OSF repository.

#### Design

In this simulation study, we used a model with two latent variables, $\eta _1$ and $\eta _2$, and their interaction as predictors of the outcome variable *Y* in a Poisson regression model. The linear predictor was:

$$\begin{aligned} \pi _i = \beta _0 + \gamma _1 \cdot \eta _1 + \gamma _2 \cdot \eta _2 + \gamma _{12} \cdot \eta _1 \cdot \eta _2 \end{aligned}$$

where we are particularly interested in the estimation of the product term coefficient $\gamma _{12}$.

The latent variables were simulated as standard bivariate normally distributed with a correlation of $r=.3$ and measured with three indicators each. We also computed sum scores as fallible substitutes of the latent variables over the three indicators, respectively. The sum scores were *z*-standardized for comparability with the latent variables. The reliabilities of the sum scores were manipulated independently by altering the measurement error variances of the indicators. We investigated the six reliability combinations for the sum scores of both latent variables, considering the reliabilities of 0.7, 0.8, and 0.9 respectively. Additional design factors where the sample size ($N=100,~200,~500,~1000$) and the size and direction of the interaction parameter ($\gamma _{12}=-0.3,~0,~0.3$)

We estimated the model with both a LV-CRM (where the means of the latent variables were fixed to 0 and the variances to 1) and a GLM (with *z*-standardized sum scores) and investigated and compared the bias and efficiency of the estimated product term parameter $\hat{\gamma }_{12}$ (or $\hat{\beta }_4$ in the GLM, respectively).

**Fig. 4:**
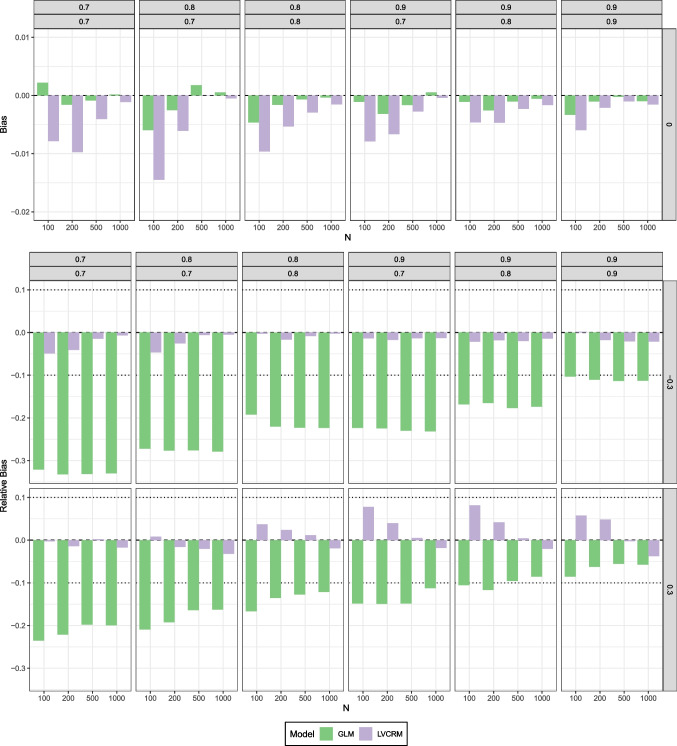
Simulation study 1: (Relative) bias of estimated product term coefficients in the LV-CRM (*purple*) and the GLM (*green*). The *upper panel* shows bias for conditions with $\gamma _{12}=0$, the *lower panel* shows relative bias for conditions with $\gamma _{12}\ne 0$. Columns reflect the six combinations of reliabilities of the sum scores, rows reflect the size and direction of the product term coefficient, *x*-axis reflect sample size *N*

#### Results

##### Convergence

We ran the simulation with $R=1000$ replications including non-converged solutions first in order to examine the convergence behavior of both approaches. Both approaches yielded convergence rates of virtually 100% in this simulation. Only in six out of 72 conditions was there one out of 1000 replications where the LV-CRM did not converge. These six conditions had a positive product term coefficient in common, but were unsystematic regarding the other design conditions (i.e., large and small sample sizes, high and low reliabilities). Thus, convergence seemed to be no issue for the specified model in sample sizes from $N=100$ upwards.

##### Bias

The following analyses are based on a second run of the simulation with $R=1000$ replications *excluding* non-converged solutions, that is, if one of both models did not converge the replication was repeated until both models converged.

We investigated the bias of the estimated product term coefficient $\hat{\gamma }_{12}$ in the LV-CRM and $\hat{\beta }_4$ the GLM, respectively. The results are presented (a) as bias for cases where the true parameter $\gamma _{12}=0$ and (b) as relative bias for cases where the true parameter was non-zero, i.e., $\gamma _{12}\ne 0$. The results are illustrated in Fig. 4 Both models yielded very small biases in conditions where the true parameter $\gamma _{12}$ was zero. For the GLM, the bias ranged between $-0.006$ and 0.002. The largest negative bias of about $-0.015$ was found for the LV-CRM in a condition with low reliabilities (both 0.7) and a small sample size of $N=100$. The upper bound of the bias for the LV-CRM was 0.000. Overall, the LV-CRM tended to underestimate the true parameter more than the GLM approach. This is not surprising, as measurement error is expected to attenuate the estimated regression coefficients of a GLM towards zero and in conditions with a true coefficient of zero, the attenuation is ’favorable’ for the estimation of this zero.

The unfavorable effects of attenuation become clear, when looking at the relative bias of the estimated product term coefficient in scenarios where the true parameter is not zero. The relative bias of the estimated product term parameter in the GLM approach ranged between $-5.6 \%$ and $-33.2 \%$. That is, even in scenarios with highly reliable score variables (both .9), we found at least 5% underestimation. If at least one predictor had a reliability of .8 or lower, the underestimation was about 10% or more. On the other hand, the LV-CRM performed better and relative bias ranged between $-4.9\%$ and $8.2\%$. Interestingly, overestimation of the product term coefficient occurred in scenarios with positive product term coefficient, highly reliable score variables, and small sample sizes. Overall, the LV-CRM provided more accurate estimates (i.e., less bias) for the product term coefficient than the GLM. For sample sizes of $N=200$ or larger, the LV-CRM yielded relative bias below $\pm 5 \%$ under all conditions.

##### Relative efficiency

**Fig. 5:**
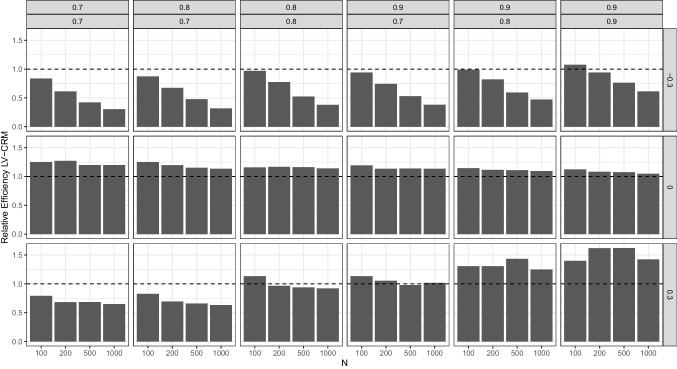
Simulation study 1: Relative efficiency of estimated product term coefficient in the LV-CRM compared to GLM (i.e., RMSE of LV-CRM divided by the RMSE of the GLM). Columns reflect the six combinations of reliabilities of the sum scores, rows reflect the size and direction of the product term coefficient, *x*-axis reflect sample size *N*

The results for the relative efficiency of the LV-CRM compared to the GLM (i.e., ratio of the respective RMSE) are presented in Fig. 5. In conditions with a true product term coefficient of $\gamma _{12} = 0$, the relative efficiency of the LV-CRM approach compared to the GLM approach ranged between 1.048 and 1.270. That is, the RMSE of the LV-CRM approach is about 4.8–27% higher than that of the GLM. Again, that is not surprising given the ’favorable’ effect of the attenuation bias in these conditions.

**Fig. 6:**
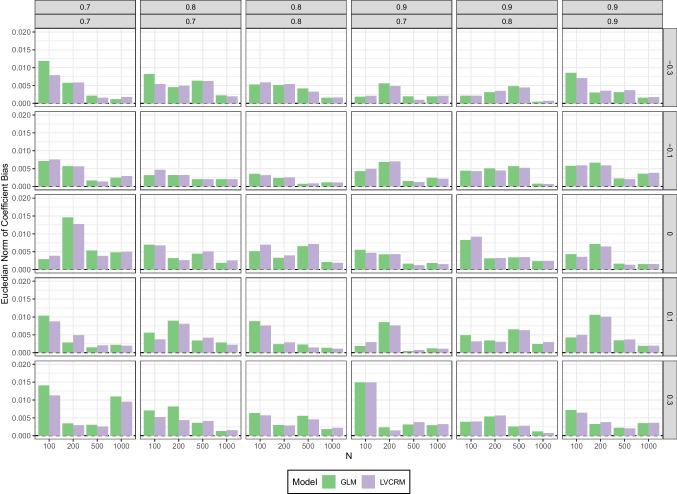
Simulation study 2: Spill-over effect $S(\hat{\beta }_1, \hat{\beta }_2, \hat{\beta }_3)$ of estimated regression coefficients in the LV-CRM and in the GLM. Columns reflect the six combinations of reliabilities of the sum scores, rows reflect the size and direction of the product term coefficient, *x*-axis reflect sample size *N*

In conditions with a true interaction parameter of $\gamma _{12} \ne 0$, the relative efficiency ranges from 0.304 to 1.622. As can be seen in Fig. 5, the LV-CRM is typically more efficient in scenarios with a negative interaction parameter, especially with larger sample sizes. With a positive product term coefficient, the LV-CRM is typically more efficient if at least one predictor has a reliability of 0.7 or both reliabilities were .8 (with few exceptions in sample sizes of $N=100$). However, with increasing reliability of the fallible score variable the LV-CRM was less efficient than the GLM.

### Simulation study 2

In the second simulation study, we extended the design of our first study in two regards: First, we investigated whether attenuation bias can have a spill-over effect on other regression coefficients, for instance, those of perfectly reliable predictors. Second, we examined whether the bias reduction in the LV-CRM approach also comes with improved statistical inferences, for example, an increase of power to detect non-zero product term coefficients. Thus, we computed 95% confidence intervals (CIs) and the empirical detection rate for each condition.

#### Design

The simulation design followed our first simulation study with a few additions: First, three additional manifest and perfectly reliable predictors were added to the regression model. These predictors were generated as independent from each other and from the latent variables. This was done to investigate potential spill-over effects of the fallible score variables.

The linear predictor was

$$\begin{aligned} \pi _i= &  \beta _0 + \beta _1 \cdot x_{1i} + \beta _2 \cdot x_{2i} + \beta _3 \cdot x_{3i} + \gamma _1 \cdot \eta _1 \\ &  + \gamma _2 \cdot \eta _2 + \gamma _{12} \cdot \eta _1 \cdot \eta _2 \end{aligned}$$

Second, standard errors, confidence intervals, and the empirical detection rate for the interaction parameter $\gamma _{12}$ were computed. Third, the random component was chosen as negative binomial instead of a Poisson distribution. This is a more realistic scenario, as it incorporates additional variance in the outcome not explained for by the predictors (which is usually the case in applied settings), but the estimation is slightly more demanding. It is also closely related to our empirical example below, where we use a negative binomial regression model.

#### Results

##### Spill-over effect

We used the Euclidean norm of the biases of the three regression coefficients of the observed covariates (i.e., $B(\hat{\beta }_1)$, $B(\hat{\beta }_2)$, $B(\hat{\beta }_3)$) to get an overall evaluation of possible spill-over effects, that is,

$$\begin{aligned} S(\hat{\beta }_1, \hat{\beta }_2, \hat{\beta }_3) = \sqrt{B(\hat{\beta }_1)^2 + B(\hat{\beta }_2)^2 + B(\hat{\beta }_3)^2 } \end{aligned}$$

The results are summarized in Fig. 6. We also computed bias and relative efficiency of the remaining coefficients (i.e., of the latent variables and the interaction term), but do not illustrate the results here as they closely resemble our findings from the first simulation study. The complete results can be found in the OSF repository.

Overall, the results indicate no spill-over effect of the fallible score variables on the estimated regression coefficients of the perfectly reliable covariates. The spill-over effect $S(\hat{\beta }_1, \hat{\beta }_2, \hat{\beta }_3)$ ranged from 0.0004 to 0.0149 for the GLM and from 0.0007 to 0.0149 for the LV-CRM. The highest values were obtained in scenarios with mixed reliabilities (i.e., one high, one low) on both fallible scores and rather low sample sizes. However, the corresponding regression coefficients appeared virtually unbiased under all conditions.

##### Coverage and empirical detection rate

**Fig. 7:**
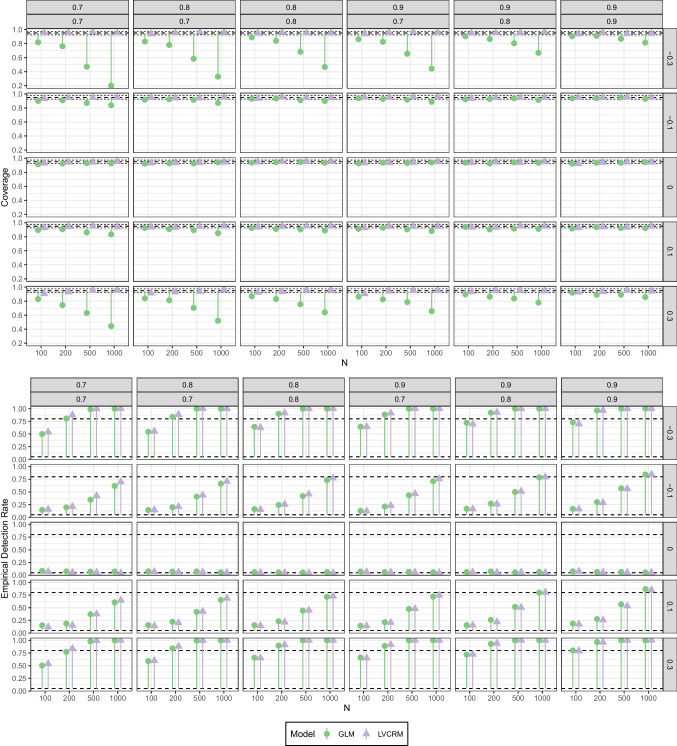
Simulation study 2: Coverage rates (*upper panel*) and empirical detection rates (*lower panel*) of estimated interaction coefficient $\hat{\gamma }_{12}$ in the LV-CRM and in the GLM. Columns reflect the six combinations of reliabilities of the sum scores, rows reflect the size and direction of the product term coefficient, *x*-axis reflect sample size *N*

We examined the coverage rate (i.e., the proportion of CIs including the true parameter value), and the empirical detection rate (i.e., the proportion of CIs not including zero) for the 95 % confidence intervals (CI) of the interaction parameter $\gamma _{12}$ estimated with both approaches (i.e., $\hat{\gamma }_{12}$ in the LV-CRM and $\hat{\beta }_6$ in the GLM). The results are summarized in Fig. 7.

In scenarios where the true interaction parameter was $\gamma _{12}=0$, both the coverage rate and the empirical detection rate (i.e., the type I error rate in these scenarios) were acceptable for both approaches. For the GLM, the actual coverage rate of the CIs ranged between 0.918 and 0.948 and the empirical detection rate between 0.052 and 0.085, respectively. For the LV-CRM, the actual coverage rate of the CIs ranged between 0.923 and 0.957 and the empirical detection rate between 0.043 and 0.077, respectively.

In contrast, in scenarios where the true product term coefficient was not zero (i.e., $\gamma _{12}\ne 0$), coverage and empirical detection rate yielded diverging results. For the GLM, the actual coverage rate of the CIs ranged between 0.199 and 0.951 and the empirical detection rate (i.e., the power in these scenarios) between 0.127 and 1.000, respectively. Notably, the coverage rate was more accurate for small interaction sizes (i.e., $\gamma _{12} = |0.1|$), but barely acceptable for larger interaction sizes (i.e., $\gamma _{12} = |0.3|$). For the LV-CRM, the actual coverage rate of the CIs ranged between 0.904 and 0.966 and the empirical detection rate between 0.121 and 1.000, respectively. Overall, the power was similar for both approaches. On average, the power was 0.7% higher for the LV-CRM, where the differences in power between the two approaches ranged from -3.9% (i.e., higher power in the GLM) to 8.2% (i.e., higher power in the LV-CRM).

**Fig. 8:**
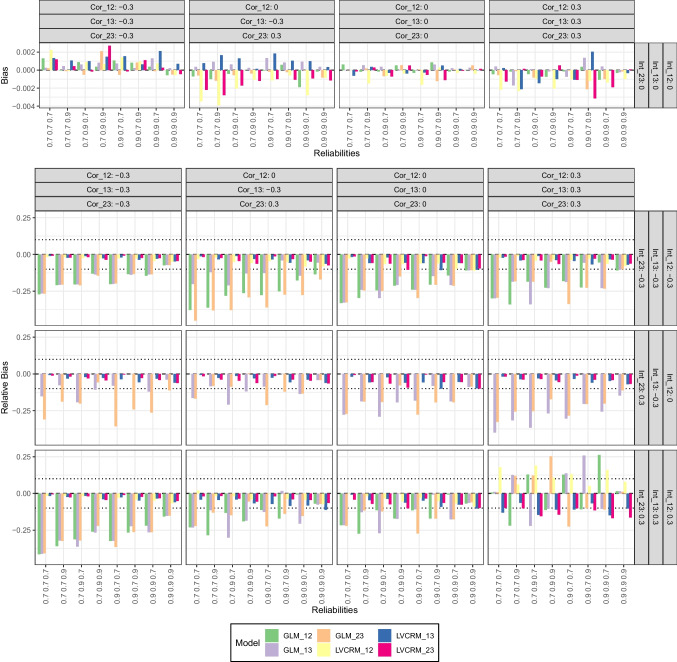
Simulation study 3: (Relative) bias of estimated interaction coefficients in the LV-CRM (*yellow*, *blue*, *magenta*) and the GLM (*green*, *purple*, *orange*). *Numbers* in the legend refer to the index of the estimated regression coefficient, for example, GLM_12 reflects the estimate of $\gamma _{12}$ in a GLM. The *upper panel* shows bias for conditions where all interaction coefficients were zero, the *lower panel* shows relative bias for the remaining conditions. In conditions with mixed coefficients, (relative) bias for $\gamma _{12}=0$ is not shown. Columns reflect the correlational patterns among the LVs, rows reflect the size and direction of the product term coefficients, *x*-axis reflects different combinations of reliabilities of the sum scores

When it comes to statistical inferences, these findings indicate that attenuation bias in the GLM is somewhat compensated for by an overconfident (i.e., too small) standard error. As a result, hypothesis testing seemed to work reasonably well, but the confidence intervals were too narrow and did often (i.e., up to 80.1%) not include the true parameter value. In contrast, the LV-CRM yielded unbiased point estimates and accurately accounted for multiple sources of uncertainty (e.g., measurement error, regression residual) resulting in wider CIs (compared to the GLM). Thus, null hypothesis testing would be expected to work with both approaches, but substantive interpretation of the CI is more reliable with the LV-CRM.

### Simulation study 3

In the third simulation study, we focused on scenarios with three latent variables and their two-fold interactions. Our goal was to investigate the extent of attenuation bias in this complex settings given different combinations of reliability, correlations, and interactional patterns among the latent variables. Similar to the first simulation study, we restricted ourselves to consider bias and relative efficiency of the estimated interaction parameters alone and did not investigate statistical inferences in order to keep the simulation computationally feasible.

#### Design

The design of the simulation study is similar to the first simulation study, but with three latent variables and their three two-fold interactions. The linear predictor was:

$$\begin{aligned} \pi _i&= \beta _0 + \gamma _1 \cdot \eta _1 + \gamma _2 \cdot \eta _2 + \gamma _3 \cdot \eta _3 + \gamma _{12} \cdot \eta _1 \cdot \eta _2\\&\quad + \gamma _{13} \cdot \eta _1 \cdot \eta _3 + \gamma _{23} \cdot \eta _2 \cdot \eta _3 \end{aligned}$$

We manipulated the following three factors: First, reliability of the sum scores of each latent variable could take the values 0.7 or 0.9, resulting in eight reliability combinations. Second, we investigated four different correlational patterns among the latent variables. These four patterns where (a) small negative correlations ($r=-.3$) among all LVs, (b) small positive correlations ($r=.3$) among all LVs, (c) no correlations ($r=0$) among all LVs, and (d) mixed correlations (negative, positive, null) among the LVs. Third, we investigated four different interactional patterns. These were (similar to the correlations) (a) small negative interaction coefficients ($\gamma =-.3$) for all LVs, (b) small positive interaction coefficients ($\gamma =.3$) for all LVs, (c) no product-term induced interaction ($\gamma =0$) for all LVs, and (d) mixed interaction coefficients (negative, positive, null) for the LVs. We did not investigate different sample sizes in this simulation, but choose a single sample size of $N=500$ throughout all conditions.

**Fig. 9:**
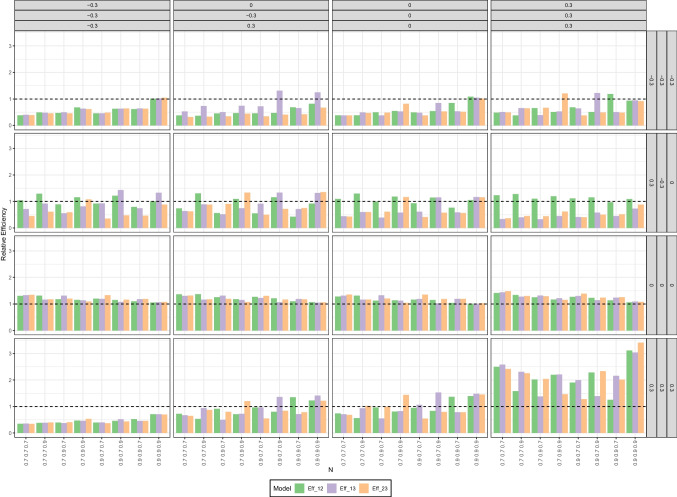
Simulation study 3: Relative efficiency of estimated interaction coefficients in the LV-CRM compared to GLM (i.e., RMSE of LV-CRM divided by the RMSE of the GLM). *Numbers* in the legend refer to the index of the estimated regression coefficient, for example, GLM_12 reflects the estimate of $\gamma _{12}$ in a GLM. Columns reflect the correlational patterns among the LVs, rows reflect the size and direction of the product term coefficients, *x*-axis reflects different combinations of reliabilities of the sum scores

#### Results

##### Bias

The results on attenuation bias are presented (a) as bias for scenarios where all interaction coefficients were zero and (b) as relative bias for non-zero interaction coefficients. An overview of the results is given in Fig. 8.

In conditions where all three interaction coefficients were zero, the bias ranged between -0.002 and 0.002 for the GLM and between -0.004 and 0.003 for the LV-CRM. As in the first simulation study, this result is not surprising given the ’favorable’ effect of attenuation bias in these conditions.

In conditions where interaction coefficients could differ from zero, we found a common pattern of relative bias in most conditions, but with a notable exception (i.e., all correlations and interactions positive), which we will discuss separately. The common pattern shows a substantial relative bias for all three estimated interaction coefficients in the GLM (between -0.451 and 0.016), but comparably low relative bias for the estimated interaction coefficients for the LV-CRM (between -0.112 and 0.020).

However, in conditions with positive correlations among the LVs and three positive product term coefficients, a rather unsystematic pattern of relative bias occurred – as displayed in Fig. 8. Here, relative biases in both directions were observed, that is, between -0.225 and 0.261 for the GLM and between -0.169 and 0.190 for the LV-CRM. In order to examine if this pattern was caused by the medium sample size and possible estimation issues, we re-run these conditions with a larger sample size of $N=2000$. However, we found the same pattern again. We are not sure why the relative bias behaves so differently under these conditions, but suspect an unfavorable combination of multicollinearity of the latent variables and their interaction terms, measurement error, and rather steep conditional effects (i.e., simple slopes) leading to highly dispersed outcome values. However, it shows that attenuation bias can have rather unexpected effects in complex scenarios involving multiple latent variables.

##### Relative efficiency

The results for the relative efficiency of the estimated interaction coefficients in the LV-CRM compared to the GLM were similar to our findings from simulation studies 1 and 2 and are therefore not discussed in detail again. An overview is given in Fig. 9 and the complete results are available from the OSF repository.

## Empirical example

We provide an empirical example from clinical psychology to illustrate how the LV-CRM framework can be applied to model count regressions with latent interactions in applied settings. Wilker et al. (2017) examined the effects of trauma load, mental defeat, and their interaction on symptom severity (i.e., incidence of symptoms) of posttraumatic stress disorder (PTSD) and dissociation in Ugandan war survivors.

### Theoretical background

The experience of traumatic events such as war, torture, sexual violence, accidents, or natural disasters can lead to the development of PTSD. The disorder is characterized by intrusive re-experiencing of the traumatic events, avoidance of trauma reminders, persistent alterations of mood and cognition, and a state of elevated arousal (American Psychiatric Association, 2022). In addition to these symptoms, survivors of multiple and interpersonal trauma are at elevated risk to show dissociative symptoms, which include feelings of derealization (e.g., feeling as if the own experience is not real), depersonalization (e.g., feeling as if being outside the own body), dizziness, and an incapability to move (Schauer & Elbert, 2010; Vermetten & Spiegel, 2014).

Importantly, after a single or few traumatic events, the majority of individuals do not develop trauma-associated psychopathology (Kessler et al., 2005). Whether an individual will develop mental health symptoms after a traumatic event largely depends on individual risk and resilience factors as well as on trauma-related predictors (Kessler et al., 2021). However, research from post-conflict settings indicates that with an increasing number of different types of traumatic events (termed traumatic load) almost every individual will develop mental health symptoms, and individual risk and resilience factors only play a subordinate role (Neuner et al., 2004; Wilker et al., 2015).

Peritraumatic cognitive processes, referring to thoughts which occur at the time of the trauma, have been identified to influence both the memory and the appraisal of the traumatic event. Therefore, they represent risk factors for trauma-associated psychopathology and important targets for trauma-focused interventions which aim at the modification of trauma memories and associated negative cognitions. One important peritraumatic cognitive process is termed mental defeat (Kleim, Ehlers, & Glucksman, 2012) and refers to a loss of mental resistance and human dignity during the trauma (Dunmore, Clark, & Ehlers, 1999, 2001). The experience of mental defeat during a trauma is associated with the development of permanent negative cognitions about the self (e.g. “I am weak” or “I am destroyed”) and the world (e.g., “I can trust nobody”), which are known to be important symptoms of PTSD. At the same time, they lead to increased avoidance of trauma-associated memories and thereby lead to the maintenance and chronification of psychopathology (Dunmore et al., 2001; Ehlers et al., 1998).

While there is a lot of evidence indicating that the peritraumatic cognitive process of mental defeat is a central risk factor for PTSD in individuals from relatively peaceful, industrialized countries, research from post-conflict settings on mental defeat was completely lacking. Since the burden of PTSD is much higher in post-conflict settings compared to industrialized countries (Charlson et al., 2019), research from this context is urgently needed to better understand factors central to trauma-associated psychopathology and its treatment in this context. Therefore, Wilker et al. (2017) conducted a study to investigate whether mental defeat would be an important predictor of PTSD and dissociative symptoms in a post-conflict population from northern Uganda. In more detail, they investigated the interplay of trauma load and mental defeat on PTSD risk, PTSD symptoms, and dissociative symptoms. Because previous research showed that individual predictors become less important at higher levels of trauma load, potential trauma load $\times$ mental defeat interaction effects were of particular interest to the study.

### Method

The description of the methods is taken from Wilker et al. (2017, pp. 3–5). For the complete methods, the reader is referred to the original article.

#### Sample

Data collection took place in villages of Nwoya district in northern Uganda. This area was severely affected by the war between the Lord’s Resistance Army (LRA) rebel group and the Ugandan governmental forces, which lasted almost two decades. The atrocities committed during this war included forced recruitment and abductions of children and young adults, killings, mutilations, and sexual offenses. Data collection took place in 2013, 8 years after the cease-fire agreement between the LRA and the governmental troops in 2005. The final sample of $N=227$ was 54% female, with a mean age of 33.29 (SD = 10.56, range = 18–62).

#### Measures

Trauma exposure was assessed by means of a 62-item event list. This event list comprised general traumatic experiences (e.g., natural disasters, accidents), war-related traumatic events (e.g., being close to combat), as well as events specific for the LRA conflict (e.g., being forced to kill somebody by the LRA). We calculated the number of different traumatic event types experienced to assess the amount of trauma exposure (traumatic load). As previously shown in the same sample, the retest reliability of this variable was $r = .82$ (Wilker et al., 2015) and, thus, was treated as a latent predictor using a single indicator approach in our analysis.

The extent of mental defeat was assessed for the worst traumatic event using the Mental Defeat Questionnaire (MDQ) in the form of an interview (Dunmore et al., 1999, 2001). The MDQ comprises 11 unipolar items (e.g., “I lost any will-power”, “I felt destroyed as a person”) and requires responses on a five-point Likert-type scale ranging from not at all to very strong. The MDQ showed a good internal consistency in the present sample (Cronbach’s $\alpha = .89$) and was modeled as a latent predictor using a multiple indicator approach in our analysis.

The main outcome of our analysis were dissociative symptoms assessed by means of the Shutdown Dissociation Scale (Shut-D; Schalinski, Schauer, & Elbert, 2015). The Shut-D includes 13 unipolar items (e.g., “Have you fainted?” “Have you felt like you were outside of your body?” “Have you felt suddenly weak and warm?” “Have you felt nauseous? Have you felt as though you were about to throw up? Have you felt yourself break out in a cold sweat?”) investigating current bodily dissociative symptoms for the past 6 months. Participants were requested to answer on a four-point scale ranging from 0 (*never*) to 3 (*several times a week*). Thus, the scale score acts as a proxy of the incidence of dissociative symptoms and behaves similarly as a count variable, that is, the lower bound represents zero symptom occurrences, the variable only takes non-negative integer values, and a certain amount of heteroscedasticity is present. Thus, Wilker et al. (2017) handled the outcome as a count variable. The Shut-D showed a high internal reliability in the present study (Cronbach’s $\alpha = .91$).

### Statistical analysis

Wilker et al. (2017) compared models of varying complexity (i.e., with and without including the covariates age, sex, and age at worst event and with and without considering potential trauma load - mental defeat product terms). In this study, in order to investigate the differences between the GLM and the LV-CRM, we calculated the full model. Accordingly, our model included the main effects of sex, age, and age at worst event. Further, trauma load, mental defeat as well their interaction were included as predictors in the regression models.

**Table 2:** Estimates of regression coefficients and standard errors from GLM and LV-CRM for the empirical example

	GLM				LV-CRM			
Covariate		Estimate	SE	*p*		Estimate	SE	*p*
Intercept	$\hat{\beta }_0$	1.812	0.389	$< 0.001$	$\hat{\beta }_0$	1.830	0.401	$< 0.001$
Sex	$\hat{\beta }_1$	$-0.349$	0.231	0.130	$\hat{\beta }_1$	$-0.316$	0.240	0.187
Age	$\hat{\beta }_2$	$-0.023$	0.017	0.180	$\hat{\beta }_2$	$-0.029$	0.018	0.111
Age at worst event	$\hat{\beta }_3$	0.010	0.020	0.613	$\hat{\beta }_3$	0.018	0.021	0.397
MDQ	$\hat{\beta }_4$	0.675	0.131	$< 0.001$	$\hat{\gamma }_1$	0.779	0.172	$< 0.001$
Trauma Load	$\hat{\beta }_5$	0.543	0.136	$< 0.001$	$\hat{\gamma }_2$	0.698	0.193	$<0.001$
MDQ : Trauma load	$\hat{\beta }_6$	$-0.253$	0.141	0.073	$\hat{\gamma }_{12}$	$-0.481$	0.199	0.016

#### Negative binomial regression

As in the original study by Wilker et al. (2017), we estimated a negative binomial regression. That is, the outcome variable $Y_i$ (i.e., the Shut-D score) is linked to the linear predictor with a log link and is assumed to follow a negative binomial distribution.

The linear predictor in this model was

$$\begin{aligned} \pi _i =&\beta _0 \!+\! \beta _1 \cdot \text {Sex}_i \!+\! \beta _2 \cdot \text {Age}_i \!+\! \beta _3 \cdot \text {(Age at Worst Event)}_i \\&+ \beta _4 \cdot \underset{\text {Trauma Load}}{\underbrace{\text {TLS}_i}_{\text {Standardized Score}}} + \beta _5 \cdot \underset{\text {Mental Defeat}}{\underbrace{\text {MDQ}_i}_{\text {Standardized Score}}} \\&+ \beta _6 \cdot \text {TLS}_i \cdot \text {MDQ}_i \end{aligned}$$

#### LV-CRM

In addition, we used the LV-CRM framework to carry out the same analysis, but including a measurement model for the latent trauma load ($\eta _{\text {TL};i}$) and mental defeat ($\eta _{\text {MD};i}$) variables in order to account for measurement error:

$$\begin{aligned} \begin{pmatrix} \text {MDQ}_{1;i} \\ \text {MDQ}_{2;i}\\ \text {MDQ}_{3;i}\\ \text {MDQ}_{4;i}\\ \text {MDQ}_{5;i}\\ \text {MDQ}_{6;i}\\ \text {MDQ}_{7;i}\\ \text {MDQ}_{8;i}\\ \text {MDQ}_{9;i}\\ \text {MDQ}_{10;i}\\ \text {MDQ}_{11;i}\\ \text {TLS}_{i} \end{pmatrix}&= \begin{pmatrix} \nu _1 \\ \nu _2\\ \nu _3\\ \nu _4\\ \nu _5\\ \nu _6\\ \nu _7\\ \nu _8\\ \nu _9\\ \nu _{10}\\ \nu _{11}\\ \nu _{12} \end{pmatrix} + \begin{pmatrix} \lambda _1 & 0 \\ \lambda _2 & 0\\ \lambda _3 & 0\\ \lambda _4 & 0\\ \lambda _5 & 0\\ \lambda _6 & 0\\ \lambda _7 & 0\\ \lambda _8 & 0\\ \lambda _9 & 0\\ \lambda _{10} & 0\\ \lambda _{11} & 0\\ 0 & \lambda _{12} \end{pmatrix} \cdot \begin{pmatrix} \eta _{\text {MD};i} \\ \eta _{\text {TL};i} \end{pmatrix} + \begin{pmatrix} \epsilon _1 \\ \epsilon _2\\ \epsilon _3\\ \epsilon _4\\ \epsilon _5\\ \epsilon _6\\ \epsilon _7\\ \epsilon _8\\ \epsilon _9\\ \epsilon _{10}\\ \epsilon _{11}\\ \epsilon _{12} \end{pmatrix} \end{aligned}$$

Both scales were fixed to a mean of zero and a variance of one and, consequently, all intercepts and loadings as well as the latent covariance were estimated freely.

Note that we modeled trauma load $\eta _{\text {TL};i}$ using a fixed-reliability single indicator approach as proposed by Savalei (2019) for two reasons: First, trauma load is not a traditional psychometric variable, but the items reflect different traumatic event types. The items are not meant to measure a common factor and are likely to be uncorrelated to some extent (i.e., experiencing a natural disaster is not necessarily correlated to having an accident). Thus, a multiple indicator approach would not have been appropriate. Second, the retest reliability of 0.82 (Wilker et al., 2015) indicates that trauma load cannot be assessed exactly, meaning there is some kind of measurement error involved. According to our simulation studies, we would expect a substantial attenuation bias on the product term coefficient given this level of reliability and, thus, explicitly controlling for this measurement error seems warranted. In order to fix the reliability of trauma load to 0.82, we constrained its measurement error variance to:

$$\begin{aligned} \text {Var}(\epsilon _{12}) = \lambda _{12}^2 \cdot \left( \frac{1}{\text {Rel}_{\text {TLS}}} - 1 \right) \end{aligned}$$

where $\text {Rel}_{\text {TLS}} = 0.82$. Then, the linear predictor for the LV-CRM was

$$\begin{aligned} \pi _i =&\beta _0 + \beta _1 \cdot \text {Sex}_i + \beta _2 \cdot \text {Age}_i + \beta _3 \cdot \text {(Age of Worst Event)}_i \\&+ \gamma _1 \cdot \underset{\text {Trauma Load}}{\underbrace{\eta _{\text {TL};i}}_{\text {Latent Variable}}} + \gamma _2 \cdot \underset{\text {Mental Defeat}}{\underbrace{\eta _{\text {MD};i}}_{\text {Latent Variable}}} + \gamma _{12} \cdot \eta _{\text {TL};i} \cdot \eta _{\text {MD};i} \end{aligned}$$

where the standardized test scores for mental defeat and trauma load are replaced with the corresponding latent variables.

**Fig. 10:**
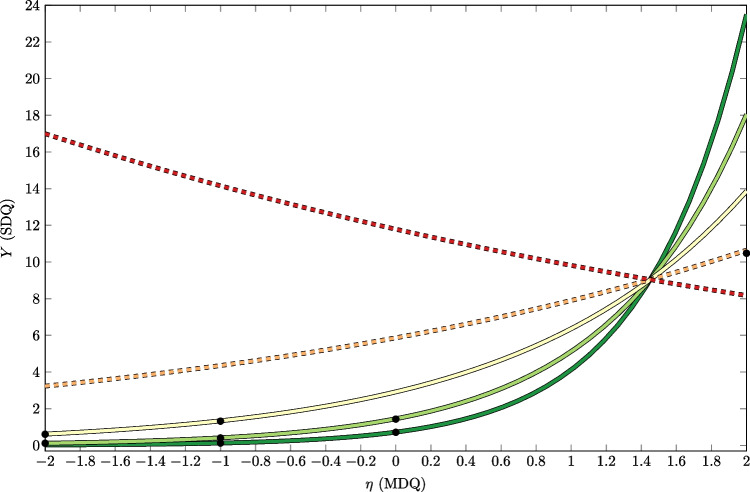
Conditional regressions for the relation between latent mental defeat (MDQ) and dissociative symptoms (SDQ) given several values of trauma load (at 2 SD below mean in *dark green*; at 1 SD below mean in *light green*; at mean in *yellow*; at 1 SD above mean in *orange*; at 2 SD above mean in *red*). *Black dots* indicate value combinations where the interaction effect $\zeta$ is significant

### Results

In a first step, we inspected and compared the estimated regression coefficients from both models. Table 2 shows the regression coefficients, their standard errors, and *p* values of the estimated GLM and the LV-CRM, respectively. As can be seen, the estimated coefficients for trauma load, mental defeat, and their interaction are larger if measurement errors are considered, as in the LV-CRM. These results are in line with our simulation results on attenuation bias and illustrate that the GLM is likely to underestimate the true parameter effects even if the reliability of the latent variables is relatively high.

Notably, the coefficient of the product term of trauma load and mental defeat was not significant in the GLM. Therefore, Wilker et al. (2017) identified a main effect model as the most parsimonious model with the best data fit and reported their results from this model. By contrast, the LV-CRM was able to identify a significant coefficient for the product of trauma load $\times$ mental defeat. Note that interaction effects are likely to be present in both models because of the phenomenon of natural interaction, that we discussed above. However, the significance of $\hat{\gamma }_{12}$ in the LV-CRM indicates that the product term of the latent variables can help explain additional complexity of the interaction pattern.

In a second step, we looked at the specific interaction effects, that is, we computed $\zeta$ from Eq. 1 with respect to $\eta _{\text {MD}}$ and $\eta _{\text {TL}}$. Figure 10 shows conditional regression plots, that is, the regression of the dissociative symptoms *Y* on mental defeat $\eta _{\text {MD}}$ conditional on values of trauma load $\eta _{\text {TL}}$. The illustration is similar to simple slopes in OLS regression, but shows non-linear relationships. The color indicates the extend of trauma load (green to red); the latent variable $\eta$ (MDQ) has a standardized scale, that is, mean of 0 and standard deviation of 1. The covariates age, sex, and age at worst event were fixed to their means. If trauma load was below the average or at the average (green and yellow lines; 1 and 2 SD below average and average, respectively), there was a (strong) positive association between mental defeat and dissociative symptoms. However, the association declines at an average trauma load and above average (yellow and orange line), and changes sign at 2 SD above average.

**Table 3:** Estimated interaction effects and confidence intervales at selected values for empirical example

		$$\eta _{\text {MD}}=$$				
		$-2$	$-1$	0	1	2
$\eta _{\text {TL}}=$	2	$-13.162$	$-9.723$	$-7.068$	$-5.030$	$-3.477$
		$[-71.977,45.654]$	$[-42.637,23.191]$	$[-22.781,8.644]$	$[-10.131,0.071]$	$[-6.982,0.029]$
	1	0.041	$-0.560$	$-1.580$	$-3.242$	$\mathbf {-5.864^*}$
		$[-2.406,2.487]$	$[-3.856,2.737]$	$[-5.433,2.272]$	$[-7.224,0.741]$	$[-11.099,-0.630]$
	0	$\mathbf {0.492^*}$	$\mathbf {0.576^*}$	0.178	$-1.959$	$-9.400$
		[0.170, 0.814]	[0.194, 0.956]	$[-0.649,1.005]$	$[-5.884,1.967]$	$[-26.140,7.380]$
	-1	$\mathbf {0.186^*}$	$\mathbf {0.408^*}$	$\mathbf {0.569^*}$	$-1.052$	$-14.488$
		[0.100, 0.272]	[0.244, 0.572]	[0.162, 0.976]	$[-4.818,2.713]$	$[-53.316,24.341]$
	-2	0.053	$\mathbf {0.196^*}$	$\mathbf {0.523^*}$	$-0.424$	$-21.830$
		$[-0.006,0.111]$	[0.108, 0.285]	[0.238, 0.807]	$[-3.764,2.915]$	$[-98.656,54.996]$

*Note.* Estimated interaction effects $\zeta$ and corresponding 95% confidence intervals (in parentheses) for different values of $\eta _{\text {MD}}$ and $\eta _{\text {TL}}$. The covariates sex, age, and age at worst event were fixed at their respective means. Bold indicates values significant at a 5% $\alpha$-level

**Fig. 11:**
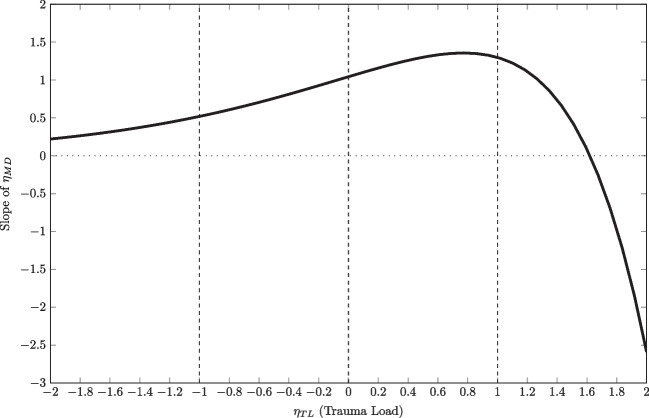
Slope of $\eta _{\text {MD}}$ for the relation between latent mental defeat (MDQ) and dissociative symptoms (SDQ) depending on $\eta _{\text {TL}}$ at $\eta _{\text {MD}}=0$ and remaining covariates at their means. *Vertical lines* indicate where the interaction effects (i.e., slopes of the slopes curve) were computed

We computed the interaction effect $\zeta$, to gain more information if and how much the slopes of the regression of dissociative symptoms on mental defeat change due to the trauma load. The results are presented in Table 3. Again, we fixed the covariates age, sex, and age at worst event to their means. We can see, that the (instantaneous) positive change in slopes due to trauma load is especially significant for values below and at average on both latent variables. This means that for persons with below or at average values of mental defeat the relationship to dissociative symptoms depends on the amount of trauma load, at least if trauma load is also below or at average. Vice versa, the relationship between mental defeat and dissociative symptoms does not (significantly) depend on trauma load, if trauma load is above average.

These results provide a first glimpse into the highly complex interactional patterns. For example, the slope of mental defeat depending on trauma load for a person with an average value of mental defeat (i.e., $\eta _{\text {MD}}=0$) is illustrated in Fig. 11. We reported the interaction effect (i.e., slope of the slope curve) from representative values of the trauma load variables (i.e., slopes where vertical lines intersect with the slope curve), but there are alternative techniques to summarize the interactional pattern. However, such techniques (e.g., average marginal/interaction effect) have to be adapted for models with latent variables.

### Discussion of empirical example

Previous research showed that at higher levels of trauma load, the interindividual variability in trauma-associated symptoms decreases and individual risk factors may only play a subordinate role (Kolassa et al., 2010; Mollica, McInnes, Pool, & Tor, 1998; Neuner et al., 2004; Wilker et al., 2015). This should be reflected by significant interactions between risk factors, such as peritraumatic mental defeat, and trauma load on the outcome variable. While this effect was only present at a trend level when employing classical negative binomial regression models, the novel method introduced in this paper allowed us to discover such interaction effects.

At the same time, the strong importance of both trauma load and mental defeat as predictors of Shutdown dissociation were replicated by the novel analyses. Due to the de-attenuation, the effects were even stronger than reported in the original analyses.

## Discussion

In psychology and the social sciences, interactions between latent predictors in count regressions are often of interest. While it is well known that using fallible scores and not accounting for measurement error generally leads to attenuation bias in the estimated regression coefficients (Carroll et al., 2006), the extent of these effects has not been previously studied for count regression models. In this paper, we introduced the latent variable count regression model (LV-CRM) framework. We examined its performance regarding point estimation as well as statistical inferences using simulation studies and illustrated its use in an empirical example from clinical psychology.

In our simulation studies, we found that severe attenuation bias (i.e., relative bias below $-10 \%$) can occur for the product terms even if the fallible scores had considerably high reliabilities (i.e., both 0.9). For non-zero product term coefficients, the estimated parameters from the GLM were attenuated up to $-33 \%$. Attenuation bias this high was observed both in scenarios with two and three latent variables and their respective interactions. In contrast, the LV-CRM yielded virtually unbiased estimates under most conditions and provided de-attenuated point estimates. In our third simulation study, we found a notable exception to this rule: In scenarios with three positively correlated latent variables and three positive product term coefficients, the relative bias fluctuated rather unsystematically for both the GLM and the LV-CRM with larger biases for the newly proposed approach. For product term coefficients of zero, however, both approaches worked equally well, with a slight advantage for the GLM due to the attenuation bias.

With regard to the relative efficiency of the point estimates, we found similar results. That is, for scenarios with a product term coefficient of zero the GLM was slightly more efficient, as the attenuation bias has a ’favorable’ effect in this case. In scenarios with non-zero product term coefficients, however, the LV-CRM was often considerably more efficient than the GLM, especially if reliabilities of 0.8 or below were involved.

Our second simulation study also investigated statistical inferences for the GLM and the LV-CRM. Interestingly, we found that the empirical detection rates (i.e., type I error rates and power) were acceptable and on the same level for both approaches. In the GLM, the biased point estimates are compensated by overconfident standard error estimates, that is, even though the point estimates are systematically closer to zero, the confidence intervals are too narrow and therefore do not necessarily include the zero too often. In contrast, the coverage rates were often poor for the GLM, especially in scenarios with larger product term coefficients. That is, the confidence intervals were often too narrow to include the true product term parameter, leading to coverage rates down to 19.9%. The LV-CRM, however, yielded accurate coverage rates under all conditions.

### Limitations and extensions

The partial derivative framework by Kim and McCabe (2022) offers a modern technique to quantify interaction effects in non-linear models, but the adaption of this framework to latent variable models can be challenging. In the empirical example, we used this framework to report interaction effects at the means of the covariates and at selected points of the latent variables. However, estimating individual interaction effects and then computing aggregates of them (e.g., average interaction effect) is not straightforward for latent variable models because the individual values of the latent variables are unobserved. Computing an average interaction effect would in this case require a mixed averaging procedure (i.e., sample average for observed predictors and integration techniques for latent predictors) or additional distributional assumptions for the observed predictors. Thus, extending the partial derivative framework for latent variable models is an important task for future research.

Wald test-based statistical inferences for estimated parameters in a SEM (e.g., for a product term coefficient) are not invariant against different methods of scaling the latent variables (Gonzalez & Griffin, 20001). In both the simulations studies and the empirical example, we identified the latent variables as standard normal scales, which performed well in terms of power and efficiency in previous studies (Klopp, & Klößner, 2021). Additionally, in the simulation studies, the data-generating model exactly matched our identification method. In the empirical example, however, a different scaling method would affect the standard errors of the estimated parameters and, thus, also the interaction effects, which are functions of these. While single parameters can alternatively be tested with a likelihood-ratio test (Gonzalez & Griffin, 20001), we are not aware of an alternative for the interaction effects. Thus, critically reflecting the choice of scale and comparing results between scaling methods seems warranted.

A well-known limitation of the marginal maximum likelihood approach is the computational burden, which becomes unfeasible if multiple latent variables ($\ge 3$) are involved. However, there exist different techniques to alleviate the computational burden (see Skrondal & Rabe-Hesketh, 2004, Ch. 6, for an accessible overview): First, numerically efficient techniques from the family of Gauss–Hermite quadratures can be used for normally distributed latent variables. Here, the integration points and weights are derived through rule-based computations. Exponential growth of the number of integration points can be drastically reduced with techniques like adaptive Gauss–Hermite quadrature or Laplace approximation. An alternative can be the use of sparse grids (Heiss & Winschel, 2008), where integration points are removed if their weight falls below a certain cutoff point, resulting in a considerably smaller grid. Second, Monte Carlo integration offers an alternative for high-dimensional integration problems as well as in situations with non-normal latent variables. Here, the integration points and weights are randomly drawn from the target distribution. In contrast to Gauss–Hermite techniques, the number of integration points does not necessarily grow exponentially and the weights are always equal to 1. Especially for non-normally distributed latent variables, this technique can be facilitated with a Gauss–Hermite rule-based importance sampling approach (Elvira et al., 2021). Third, in some cases, it is possible to reduce the dimension of numerical integration below the number of latent variables. Rockwood (2021) illustrates this in an example with five latent dimensions, where one dimension of integration suffices after a re-parameterization of the model. While this reduction technique does not generalize directly to a model with interaction terms, it can be useful in situations where only few of the latent variables are actually involved in interactions.

While the LV-CRM is an extension of both the G-SEM framework (Rockwood, 2021) and the NB-MG-SEM framework (Kiefer & Mayer, 2021a, b), it is more restrictive as these frameworks in some regards. The LV-CRM can in principle be extended to be a full generalization of the G-SEM framework. Such a generalization would include multiple outcome variables, a structural model among the latent variables, and allow for manifest covariates in the measurement model. Especially the possibility for multiple outcomes would allow for more complex regression models, for example, zero-inflated count regression models. In addition, a G-SEM model also allows for non-linear measurement models, that is, item response theory (IRT) models for categorical and count outcomes can be adapted for the measurement model (Rasch, 1960). Vice versa, modern IRT models for count models could be adapted to provide more versatile count regression models (Beisemann, 2022; Forthmann et al., 2020).

The two main differences (besides the latent interactions) between the LV-CRM and the NB-MG-SEM are: First, the NB-MG-SEM is based on a multigroup framework which allows for more modeling flexibility when it comes to group-specific effects. For example, it is possible to estimate group-specific overdispersion parameters or measurement error variances. Thus, it offers an alternative to model heterogeneity in parameters. Second, in the LV-CRM, we distinguished between stochastic and fixed observed variables. That is, manifest predictors in the LV-CRM are considered as fixed by design. The NB-MG-SEM models all observed variables as being stochastic (i.e., randomly sampled). While this distinction should have no effect on the estimation of the regression coefficients (Kiefer & Mayer, 2019), the stochastic approach additionally estimates various moments (i.e., expectation, variance, covariance) of the manifest predictors, which can be used for further analyses.

## Data Availability

The datasets generated during and/or analyzed during the simulation studies of the current study are available in the OSF repository, https://osf.io/q7knc The dataset analyzed for the empirical example of the current study are not publicly available due to data privacy laws. Because of this limitation, analysis code is illustrated with a synthetic dataset, which is also available from the OSF repository.

## Footnotes

1 It is possible to allow for quadratic terms of the latent variables (e.g., $\eta _k^2$) by changing the starting index of the second sum from $k+1$ to *k*.

## Code availability

Materials and analysis code are available at https://osf.io/q7knc.
